# Gendered differences in academic emotions and their implications for student success in STEM

**DOI:** 10.1186/s40594-018-0130-7

**Published:** 2018-09-06

**Authors:** Michael Pelch

**Affiliations:** Department of Biology, University of Washington, Box 351800, Seattle, WA 98195-1800 USA

**Keywords:** STEM, Gender, Student anxiety, Grounded theory, Emotion, Self-regulated learning

## Abstract

**Background:**

Understanding student anxiety is an important factor for broadening the gender diversity of STEM majors due to its disproportionate and negative influence on women. To investigate how student anxiety is related to other academic emotions I conducted open-ended interviews with 19 university students and analyzed the data using emergent grounded theory. Emergent grounded theory uses inductive and deductive reasoning to develop a model of cognition and human behavior.

**Results:**

Data analysis led to the development of a detailed theoretical model outlining connections among student anxiety, positive and negative academic emotions, self-regulated learning, and performance. In addition, the data highlight important emotional differences between men and women that have the potential to influence retention in STEM. Specifically, the model elaborates on the concept of a self-deprecating cycle driven by negative academic emotions and suggests that women may be more likely to become trapped in this cycle.

**Conclusion:**

The model incorporates students’ emotions as a powerful influence on performance and can be used to inform strategies aimed at changing how university students experience and deal with emotions such as student anxiety.

## Background

The President’s Council of Advisors on Science and Technology (PCAST) states that the USA will need roughly one million more science, technology, engineering, and mathematics (STEM) graduates to keep pace with the rising demand for STEM professionals (Chen and Soldner 2013). To meet that demand, it will be critical for many STEM disciplines to enact strategies aimed at reaching greater parity between the numbers of men and women completing their degree programs (NSF 2017). However, increasing the numbers of women with STEM degrees is challenging because research has shown that women lose interest and subsequently leave the STEM pipeline at higher rates than their male peers at all levels of education (Ellis et al. 2016).

A cognitive model proposed by Graham et al. (2013) characterizes why STEM-interested students stay or leave. In this model, classroom activities influence both learning and the development of students’ identity as scientists. The model describes a positive feedback loop suggesting that when students begin to learn science, they gain the confidence to start perceiving themselves as scientists; as they begin to identify as scientists, they gain the motivation to devote more effort to the activities that help them learn science (Graham et al. 2013). Because student learning, confidence, and motivation all have significant emotional components (Meyer and Turner 2002), a better understanding of the gendered differences in academic emotions and their influence on student learning could potentially help address the gendered gap in STEM persistence.

Anxiety is one of the most common, relatable, and influential emotions related to academics. According to a recently published survey (*n* = 95,761), over 23% of undergraduates stated that anxiety impacted their academic performance (American College Health Association 2014). Anxiety related to academics is common among individuals 18 years or older (Kessler et al. 2005), and its negative effects have been reported in all disciplines (Markman et al. 2011). Anxiety is described as a subjective feeling of tension, apprehension, nervousness, and worry associated with nervous system arousal (Spielberger 1980). More specifically, Vitasari et al. (2010) defined “student anxiety”—as opposed to general anxiety—as feelings, thoughts, and experiences that create apprehension during the study process or course experiences, and that subsequently influence academic performance (Khoshlessan 2017).

Female undergraduates report significantly higher levels of student anxiety than their male peers, and that trend holds regardless of grade point average (GPA) or preparedness (Chapell et al. 2005). The gendered gap in student anxiety initially appears around age nine and increases through post-secondary education (Czerniak and Chiarelott 1985). To understand the dynamics of anxiety and gender more thoroughly, Goetz et al. (2013) investigated how students perceived their anxiety and compared that to their anxiety during high-stakes testing. In a key finding, the female students in their study perceived themselves as more test-anxious than male students, even though no difference in anxiety occurred during actual testing. The researchers attributed this result to female students having less confidence than male peers in their ability to perform during high-stakes tests.

Despite the breadth of research studies, researchers have not reached a consensus regarding the mechanism responsible for gendered differences in anxiety; one possibility is emotional intelligence (EI). EI is defined as an individual’s ability to cope with and process emotions and has been shown to be a significant regulating factor for influencing emotions related to academics (Parker et al. 2004; Thomas et al. 2017). Because some studies have reported that women tend to have higher levels of emotional intelligence (EI) than their male peers (Naghavi and Redzuan 2011), gendered differences in EI could explain some of the self-reported gendered differences for student anxiety. EI has been shown to have numerous positive relationships with student performance (Parker et al. 2004). However, how EI interacts with learning is complex, and this complexity has been represented in contradictory findings where no relationship was shown between EI and performance, or where individuals with heightened EI can experience conflicting emotions. For example, higher levels of EI can make individuals susceptible to additional stress. For example, Ciarrochi et al. (2002) reported that higher emotional perception (a dimension associated with EI) can make some individuals particularly sensitive to environmental emotional stressors, causing them to experience greater degrees of struggle. Studies on the relationships between anxiety and emotional intelligence report that individuals with high anxiety often have difficulty managing their emotions and employing skills to change them (Fischer et al., 2010).

Another explanation for the male-female differences in student anxiety could be attributed to socialization with respect to expressing and dealing with emotions such as anxiety. Women are more predisposed than their male peers towards expressing issues with anxiety (Bernstein et al. 2006). Women are also more likely to receive positive reinforcement for expressing concerns about their anxious feelings while men are not (McLean and Anderson 2009). Additionally, women are more likely to employ emotion-focused coping mechanisms than men (Thoits 1987) which could partially account for their willingness to share their emotional experiences as well as an increased awareness of emotions related to student anxiety. This gendered difference in the willingness to share emotional experiences may be a particularly important confounding factor in studies employing qualitative methods. Studies have also shown that female students have a higher prevalence for emotional distress and self-derogation, both of which can have a negative impact on the course and exam performance (Cassady and Johnson 2002; Hembree 1988). Chin et al. (2017) found that female students reported higher levels of test anxiety, positive affect, and negative affect than their male peers and that higher test anxiety led to lower course grades for female participants. They also suggested that the relationship between gender and negative affect may be a significant indicator of an individual’s predisposition towards high test anxiety and poor course grades.

### Test anxiety

Anxiety related to evaluative events, or test anxiety, is the best-studied aspect of student anxiety, and female students consistently report higher levels of test anxiety than their male peers (Cassady and Johnson 2002; Goetz et al. 2013; Hembree 1988; Robichaud et al. 2003; Zeidner 2010; Zeidner 1990). Test anxiety has commonly been sub-divided into an emotional component and a worry component (Cassady and Johnson 2002; Chapell et al. 2005; Sarason 1984). The emotional component comprises the physiological symptoms associated with anxiety while the worry component, referred to as cognitive test anxiety, describes the psychological symptoms (such as diminished recall) that can impede learning and negatively impact exam performance (Cassady and Johnson 2002). This two-dimensional model predicts that learning is reduced when the emotional and worry components interact (i.e., Hembree 1988). While the two-dimensional model of test anxiety focuses on the worry and emotional components, other earlier studies have included task-irrelevant thinking and tension as crucial dimensions of test anxiety (Sarason 1984). Chin et al. (2017) integrated the worry, emotional, and task-irrelevant thinking dimensions with a fourth factor—academic-related apprehension, or tension—to account for the co-morbid relationship of test anxiety with other forms of emotional distress. They found that tension related to test anxiety was a significant factor and that the four-dimensional model explained 18% of the variance in exam performance; which is more than the 5–10% reported in studies using the two-dimensional model of test anxiety.

Numerous studies have shown a significant negative correlation between the cognitive dimension of test anxiety and exam performance and GPA (Cassady 2004; Cassady and Johnson 2002; Goetz et al. 2013; Hembree 1988; Thomas et al. 2017; Zeidner and Schleyer 1998). However, studies have also shown that test anxiety only explains 5–10% of the observed variance in exam scores (Cassady and Johnson 2002; Chapell et al. 2005; Chin et al. 2017; Karatas et al. 2013). Test anxiety is highly co-morbid with other psychological disorders, such as general anxiety and depression, both of which are also strongly correlated with diminished academic performance. For example, general anxiety has been shown to be a significant mediator and precursor of heightened test anxiety (Beidel et al. 1994; Zettle 2000). Due to this overlap, studies attempting to measure test-specific anxiety may be inadvertently measuring the broader construct of emotional distress (Ollendick et al. 2003). Emotional distress is a part of the “negative affect” dimension of the tripartite model of emotions or TME (Clark and Watson 1991). The TME highlights this substantial overlap between test anxiety and other forms of anxiety, considers both the negative and positive impact on affect, and incorporates physiological hyperarousal (Clark and Watson 1991). Chin et al. (2017) established that the negative affect portion of the TME had an indirect impact on test anxiety and confirmed the results of other studies, showing that the physiological symptoms of test anxiety have a limited role in mediating exam scores.

Theories to explain how test anxiety reduces performance center on anxious feelings and emotions inhibiting an individual’s working memory—often labeled cognitive interference (Jamieson et al. 2013; Owens et al. 2012; Tobias 1985). Other studies have presented an alternative to the cognitive interference model by proposing a skills-deficit model, where a student’s self-regulatory skillset and study behaviors can be influenced by test anxiety (Covington 1985; Pekrun et al. 2002; Ramirez and Beilock 2011; Rasor and Rasor 1998). Rasor and Rasor (1998), for example, showed that students’ preferences for poor study habits were a significant predictor of higher test anxiety. Choices of study habits, in turn, could be mediated by a students’ emotional responses to their academic situations. In addition to poor study habits, previous work has proposed that test anxiety is related to performance through students’ achievement goals (Pekrun et al. 2014), as well as scholastic resilience (Putwain and Daly 2013). Another study by Spachtholz et al. (2014) revealed that the broader category of negative affect can impede a student’s capacity for cognitive tasks but not necessarily their cognitive precision. These results provide a foundation based on interactions between testing-specific anxiety and the array of other emotions that can impact students’ academic experiences, their exam performance, and ultimately their course performance.

### Emotions related to academics

Compared to test anxiety, the broader range of academic emotions and their role in student success has not been as thoroughly investigated (Pekrun et al. 2002; Schutz and Lanehart 2007; Schutz and DeCuir 2002). Wäschle et al. (2014) described a virtuous cycle associated with positive emotions and high self-efficacy and a vicious cycle marked by negative emotions and procrastination. They also showed that these contrasting cycles impacted not only how students “felt” but also their study strategies and motivations to succeed. This connection between academic emotions and study strategies was also proposed in an earlier study by Covington (1985). In their model, they described a cycle of self-deprecation that was driven and reinforced by feelings of anxiety and poor study habits. Other researchers have tied positive emotions, such as joy and contentment, and negative emotions, such as disappointment and guilt, towards students’ self-regulated learning strategies and their tenacity to persevere through academic challenge (Pekrun et al. 2002). Pekrun et al. expanded on the relationship between emotions and academics and proposed a social-cognitive, control-value theory, which expanded on Pekrun’s earlier work, outlining a cognitive-motivational model where positive and negative emotions can either be activating or deactivating. Activating emotions like pride are feelings that motivate students to succeed, while deactivating emotions like shame are related to outcomes or the assessment of performance. The updated social-cognitive model places the feedback that students received as a significant mediator of their emotions. Additionally, the social-cognitive, control-value theory suggests reciprocal links between students’ emotions and their self-regulatory skills (Pekrun et al. 2002). This connection between emotions and self-regulation was echoed in a study by Lukes and McConnell (2014), who found that students described a significant emotional component in their motivation to study for exams, where certain strategies were employed as a method to avoid negative academic emotions.

The relationship between emotions and motivation has been well established, but that relationship often played a more minor and isolated role compared to research focusing on the connections between cognition and motivation. Pursuing the connection between emotions and the motivation to achieve, Pekrun et al. (2014) proposed that achievement goals and motivations should be viewed in concert rather than as isolated constructs. Meyer and Turner (2002) found that negative emotions were associated with lower learning goals regardless of the student’s ability; they also concluded that emotions are powerful mediators of students’ motivations to learn.

### Current study

Literature agrees that heightened student anxiety is more prevalent in women and can have a negative impact on their decision to pursue STEM degrees and careers (Udo et al. 2004). Additionally, studies have emphasized the need for research to focus on the context of emotions related to academics and academic experiences (Schutz and DeCuir 2002). As a result, student anxiety—as well as other academic emotions—should be considered an important mediating factor for improving and promoting gender diversity in STEM. The important role of academic emotions and gender in STEM led me to explore gendered differences in students’ descriptions of their academic experiences and emotions. The goal of this study was to create a broad, multi-dimensional model of students’ academic emotions grounded in the contexts of their academic experiences.

## Methods

Emotions are fluid, dynamic, and are difficult to investigate using traditional research methods (Schutz and DeCuir 2002). Studies have also suggested to analyze emotions using more holistic methods and to characterize academic emotions in a non-linear way (Scherer 2000). Consequently, the foundation of this study used an emergent qualitative investigation that combined inductive and deductive techniques. Qualitative analyses allow for both a more holistic analysis as well as having the methodological flexibility to consider a more complex, non-linear relationship among constructs. I also chose to use an emergent design because I hypothesized that students’ descriptions of their academic experiences and academic emotions would involve a variety of psychological constructs and dimensions. In fact, the role and impact of academic emotions is often difficult to fully examine by viewing it through the lens of a single emotion (Thomas et al. 2017). Schutz and DeCuir (2002) reiterate this assumption pointing out that a variable-focused quantitative approach towards investigating emotions often leads to reductionist interpretations. Therefore, using a survey designed to measure a single dimension would limit the transferability of the results and present a much narrower representation of how gendered differences are expressed throughout students’ descriptions of their experiences.

### Participant selection

Participant selection was guided by the need to have sufficient gender representation, as well as students who self-reported issues with cognitive test anxiety. Participants were purposely chosen based on their responses to the Cognitive Test Anxiety Scale (CTAS; (Cassady and Finch 2014), which consists of 17 Likert-type items. The CTAS focuses on the cognitive domain of test anxiety and participants responded to each prompt on a four-point scale from “Not at all like me” to “Very much like me”. The 17-item version of the CTAS dropped all of the reverse-scored items which distinguishes it from the original 27-item version (Cassady and Johnson 2002). I acknowledge that since the collection of these data using the CTAS that the survey has been further revised and that the newest version, labeled the CTAS-2 (Thomas et al. 2017), consisting of 24 items was not employed in this study.

The 17-item CTAS survey was administered to 1086 students taking introductory biology at a large public university in the Pacific Northwest. The demographic distribution of students in introductory biology is 62% female, 38% male, 41% Caucasian, 24% Asian, 14% multinational, 10% international, 7% Hispanic, and 2% African American (according to the university registrar database). The majority of students in introductory biology are freshman or sophomores and, due to university regulations, have not formally declared majors at the time of data collection. However, 90% of the students in this class had declared an interest in a STEM discipline or pre-medical degree during university admission. Item scores on the CTAS resulted in a Cronbach alpha of 0.93 which is considered to be a high degree of internal consistency and confirms the reliability of this instrument for the population of students in this study.

Participation in the study was opened up to the entire class roster through a mass email advertisement. From the entire course population, 70 students responded. Male and female students who volunteered for the study were stratified by their CTAS scores into bins of high (CTAS scores 45 and greater), moderate (CTAS scores 24–44), and low (CTAS scores 23 and lower) total scores to represent different levels of test anxiety. This method of characterizing different individuals along the continuum measured by the CTAS has been used on the 27-item version of the CTAS (Cassady 2004) and then further refined in the CTAS-2 (Thomas et al. 2018). However, the severity standards used in this study are not validated and do not reflect those established through exploratory latent class analysis of the CTAS-2 by Thomas et al. (2018). The intention behind the use of severity standards in this study was not to establish or compare statistically distinct groups; rather, it was used as a way to ensure that the interview sample contained participants representative of a variety of self-reported cognitive test anxiety levels based on of their responses to the 17-item CTAS.

Nineteen students who were interested in STEM degrees were chosen as the final interview participants (Table 1). Five men were finally selected because only 11 men volunteered from the entire class, and most of them either clustered in the low CTAS bin or did not respond to multiple rounds of follow-up emails. Participant selection stopped at 19 students because I had reached a study sample that was demographically similar to the larger class population, and the study sample contained both men and women with moderate and high self-reported CTAS scores. Each participant received a 20-dollar gift card as compensation for their time.

**Table 1:** List of study participants gender, ethnicity, CTAS score, and CTAS score label

Participant	Gender	Ethnicity	CTAS Score	CTAS Label
1	Female	Asian	48	High
2	Female	Asian	54	High
3	Female	Asian	59	High
4	Female	Asian	47	High
5	Female	Asian	54	High
6	Female	Asian	54	High
7	Female	Multi	53	High
8	Female	Asian	24	Moderate
9	Female	Caucasian	29	Moderate
10	Female	Multi	38	Moderate
11	Female	Multi	26	Moderate
12	Female	Caucasian	39	Moderate
13	Female	Multi	44	Moderate
14	Female	Asian	44	Moderate
15	Male	Asian	50	High
16	Male	Asian	36	Moderate
17	Male	Asian	36	Moderate
18	Male	Caucasian	41	Moderate
19	Male	Caucasian	25	Moderate

### Data collection

All 19 interviews were conducted one-on-one in an open format and were recorded using a digital audio recording device. Interviews were conducted in a variety of private rooms across campus at times and dates that were most convenient to the participants. Audio files were sent to a third-party transcription service and were subsequently checked for accuracy upon receipt. While interviews were largely unstructured, I began the interview process with a broad conceptual map for how I intended to proceed through the interviews and how I intended to prompt students to be open about their emotions and experiences. Interviews often focused on courses, exams, and university life as well as the feelings and emotions resulting from students’ experiences with those phenomena. I also wrote an interview reflection directly after completing each interview. These represented an additional record of the conversation between myself and the participants and were used in the data analysis to help determine the appropriate codes and contexts for each participant’s statements. Reflections often contained information on my opinions of the participant’s feelings during our conversations, how the dialog progressed between myself and the participant, and comments and questions about items that participants chose to focus on or avoid during the interview. The collection of interview data was approved by the University of Washington Human Subjects Division, and all participants confirmed their involvement by signing an approved consent form.

### Data analysis

I employed an emergent grounded theory approach to analyze data inductively (Cohen et al. 1969) and then used a content analysis to identify gender differences among the students’ statements. Emergent grounded theory is differentiated from the more commonly used systematic grounded theory method in that it does not use a pre-determined arrangement of categories (Charmaz 2008). Emergent grounded theory allowed me to approach the data with an open mind and to use participant statements, and the contexts of those statements, to define the results. Atlas.ti software was used to organize, evaluate, construct memos, code interview data, and create the emergent model. The first step was to read through every transcript, making notes and asking questions of the data in order to develop theoretical sensitivity. Initial reading of the transcripts was followed up by writing a detailed memo characterizing my thoughts about what each student was describing and how they viewed events in their lives, along with my general impressions of the participant’s statements. This initial phase of data analysis also included referring back to my original interview reflections to help with interpreting students’ statements. This open coding step identified approximately 2000 quotations of interest that were then grouped into tentative codes. The second phase consisted of more focused coding, where I evaluated each code with respect to its alignment with the quotations that comprised it and with all other codes. This process has resulted in the merging or division of codes. Next, conceptually similar codes were merged into preliminary code groups. Each code group and the codes assigned to it were evaluated and compared independently. Code groups were then merged or divided based on my interpretations and notes.

The final phase of coding consisted of evaluating and comparing the code groups and further combining them to create categories. Code groups represented distinct portions of students’ talk during the interviews. However, quotations within those code groups often co-occurred. This means a single quotation could be assigned more than one code and/or code groups. Consequently, code co-occurrence was used to gauge the level of association between the categories that comprised the final emergent model. Using the patterns of code co-occurrences and my interpretations of how participants talked about key topics also helped determine the semantic relationships between categories and also to inform the construction of the model.

To determine differences in gender within the model, I conducted a content analysis of the categories within the emergent themes. Specifically, I used Atlas.ti to tag quotes with a male or female identifier based on their source document. Because there were more female participants than male participants, I converted raw counts in each category to proportions by dividing by the total quotations for either male or female participants. Then, I calculated the percent difference between those proportions to represent whether male or female statements were more representative of a particular category.

### Validation

The emergent categories and theoretical model presented in this research were validated through member checking, in which study participants evaluate the results for alignment with their own experiences (Long and Johnson 2000; Strauss and Corbin 1990). Member checking was conducted through an online survey that consisted of six Likert-scale questions and an open response prompt where students were asked to explain their choices. The questions focused on determining whether or not participants of the interviews saw themselves or their experiences as being a part of the category descriptions and the emergent model. After numerous attempts to solicit participation, only six of the interview participants responded in sufficient detail to the member checking. However, those six students were representative of all three levels of test anxiety and both genders. All of the participants who responded agreed that they could identify themselves in the positive or negative academic emotions themes and confirmed that the results are a reasonable approximation of what students can experience. Additionally, all the students at agreed that they could personally identify with how the emergent model connected emotions, testing, and learning.

## Results

**Fig. 1:**
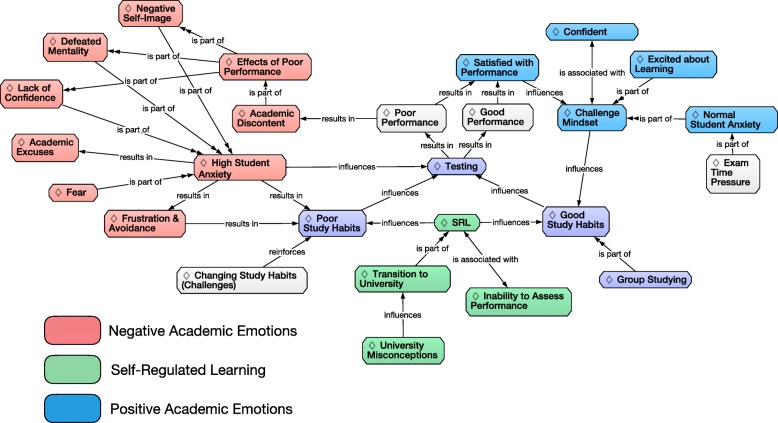
Emergent model representing the data analyzed in this study. Lines connecting and the category labels represent the interpreted semantic relationships between the linked categories

Categories that emerged from the interview data (Table 2) were connected through semantic relationships to create a model representing the study participants’ academic experiences and emotions (Fig. 1). Specifically, emergent connections among categories were synthesized with established relationships in the literature. Some of the emergent relationships—for example, the relationship between Negative Self-Image and Academic Excuses with Negative Student Anxiety—were established through co-occurrences during data analysis. Other relationships—for example, the connections between High Student Anxiety and Testing and Good Study Habits and Self-Regulated Learning (SRL)—were reflected in the data analysis of this study as well as being identified in previous research studies that used quantitative methodologies. Some of the emergent categories (i.e., Parents and Personal Interactions) are not represented in the final emergent model because they did not fit the emergent network of semantic relationships or they were representative of too little student data to confidently fit them within the network.

**Table 2:** Emergent categories with descriptions and exemplar quotations

Category	Category Description	Example Quote
Poor Study Habits	Occurrences where students describe study strategies shown to be ineffective or where students explicitly state that they know their study methods are not working	“I’d just do the reading the night before and I took notes for the meeting, and then I took the quiz for the reading quizzes.”
Course Characteristics	Descriptions, complaints or comments directed at courses and instructors	“Biology is definitely something I keep up with because I actually really, I do not want to say enjoy it, but I appreciate the structure.”
Frustration and Avoidance	Descriptions of feelings or behaviors indicative of frustration or avoidance behaviors explicitly or implicitly tied to anxiety or fear	“I don’t know how to study for this class. I’ve tried everything – office hours, studying alone, groups – that people have said to try in terms of studying, but nothing’s worked.”
High Student Anxiety	Codes qualifying the thoughts and behaviors associated with high anxiety	“Sometimes I stress myself out a lot over nothing.”
Transition to University	Comments and/or experiences about the academic differences between high school and the university	“...except in high school, they baby [sic] you everything.”
Normal Student Anxiety	Comments and codes inferred to be associated with normal levels of anxiety that are commonly experienced by most students	“...and then so it’s again motivation definitely. Obviously, you are gonna [sic] run hard if there’s a dog chasing you.”
Excuses	Codes where students blame other factors such as the class or teacher for their lack of understanding or poor performance	“I am like there must be something on the test that screws everyone over.”
Good Study Habits	Occurrences of study habits related to strategies that have been shown to improve learning	“I tried to come up with questions that they might ask me about a concept that we learned.”
Self-Regulated Learning	Codes explicitly or implicitly refer to tasks, actions, or behaviors dealing with any type of self-regulated learning or metacognition	“...the way I study really sucks. I just read through my notes, and I try to look up like practice exams, and try to do problems, but not really; sometimes I’ll just look over the answer key.”
University Misconceptions	Fallacies and odd perceptions of school, careers, faculty, or learning	“...but it’s nothing like college where it’s almost like it’s a make or break, right?”
Changing Study Habits	Students’ descriptions of the challenges or influential factors associated with altering pre-existing study habits	“Even during first quarter, exam after exam I would do the same thing.”
Time	Identifying occurrences where students comment how exam time influenced their feelings or actions during an exam	“Because I feel like there’s just so much pressure. I have to finish all of this in the given amount of time.”
Negative Self-Image	Negative perceptions of themselves or a perception strongly tied to something like grades or class performance	“I am just dumb.”
Testing	Strategies or experiences directly related to taking tests in any class	“...and so, I think I spend too much time in my own head on tests rather than just doing the material.”
Lack of Confidence	Student comments focused on a negative perception of their ability or worth	“I am an undergraduate. I have not even finished college and I am around smart people. Do I actually deserve to be here?”
Academic Discontent	Codes from students not satisfied with their previous or current academic performance	“Second exam I did not do so well. I was like below average so that was like really discouraging.”
Defeated Mentality	Codes qualifying statements as negative, defeatist, giving up, not worth the effort, or why should I try	“I do not even try to understand those parts. I know I am going to miss that, so I am not going to waste my time on it.”
Confident	Comments that show a positive outlook on their performance and see their accomplishments in positive lights	“I have gotten above average on all of them. I remember the second one, I got way above average, and that was the one I that I thought I did really poorly on, and then I ended up doing pretty good.”
Effects of Poor Performance	Statements where students describe how poor performance, both long term and short term, changes the way they behave or the way they thought about school or studying	“I hate it. It makes me so mad…”
Group Studying	Comments or experiences about studying with peers either in class or outside of class	“...if you do not understand something, there’s a good chance someone else will, and they’ll be able to explain it to you in a way that you never thought could be explained in your head.”
Inability to Assess Performance	Challenges around predicting how well they will do on exams or how well they perceive they understand the content.	“I’ll feel good about it, but then, I’ll not get a good score.”
Challenge Mindset	Codes dealing with a healthy or positive outlook on life, school, or grades	“…and I think that by giving up I would really disappoint myself.”
Higher Order Thinking	Comments oriented towards cognitive task involving analysis, evaluation or synthesis	“The material wasn’t that hard, but the tests were really hard because it required you to apply all the different parts of it to a really hard problem.”
Fear	Explicit descriptions about being afraid of something related to school or if they had an experience in school that made them feel some variety of fear	“Maybe it’s because if you ask a question, you are put on the spot kind of thing. It really scares me.”
Parents	Comments about the strong influence parental perception and desires have on their academics	“Probably my parents. Since I was young, they have been pushing, ‘Education is the most important. You should spend more of your time and energy into that.”
Excited about Learning	Positive statements about students’ attitudes towards school and learning	“Oh yeah, I love it [university]. It’s like a dream.”
Satisfied with Performance	Comments from students who are satisfied with their overall academic performance or their performance on individual courses or exams	“I think I have been doing pretty good.”
Personal Interactions	Comments elaborating on the importance on social connections with other students or faculty	“So, there was like less interaction with the teacher, and there’s a lot more people who are going on office hours, so the teacher does not really remember you.”
Preparation	Attitudes about preparing for exams	“It’s not even like some I know and some I do not. I am just not ready for this test.”
Passive Learning	Students describing passive learning behaviors and how they relate to their academic experiences	“I really like it when teachers repeat things and teach everything in class.”

### Emergent model

Similarities among the categories and relationships in Fig. 1 led to the development of three themes representing major portions of the emergent model: (1) negative academic emotions, (2) positive academic emotions, and (3) self-regulated learning. Many of the categories within the Negative Academic Emotions theme contain statements that co-occurred. For example, statements in the Frustration and Avoidance category would often co-occur with statements in Poor Study Habits as well as statements focused on High Student Anxiety; statements in High Student Anxiety co-occurred with statements coded as Lack of Confidence, Academic Excuses, and Fear. Additionally, the relationships among categories show that the Effects of Poor Performance connects a spectrum of negative academic emotions, including Negative Self-Image, Lack of Confidence, and a Defeated Mentality. These emotions also co-occurred with student talk about High Student Anxiety.

The Challenge Mindset category is the central component of the Positive Academic emotions theme. Co-occurring relationships revealed that Normal Student Anxiety is a commonly voiced aspect of students’ Challenge mindset. The Challenge Mindset category was also associated more with Good Study Habits than Poor Study Habits, as well as often being associated with confident student statements (i.e., Confidence category). The SRL (self-regulated learning) theme was placed in a central part of the emergent model summarized in Fig. 1 because, like testing, it provided a link between students’ statements in the Negative and Positive Academic emotions themes. The SRL category is also associated with students’ descriptions of their study behavior, which in turn has a direct impact on their performance.

Most of the participants in this study were first-year university students. Consequently, many of the statements that comprised the Transition to University category involved their feelings about the numerous challenges they encountered in their first quarters. Student descriptions of these challenges often involved descriptions of their limited self-regulated learning “tool-kit,” much of which involved their poor ability to self-assess learning and performance (i.e., Inability to Assess Performance category). Because many of the students discussed misconceptions and rumors about what it is like to be in a university, University Misconceptions emerged as a distinct category often co-occurring to their statements within the Transition to University category.

### Gender analysis

**Fig. 2:**
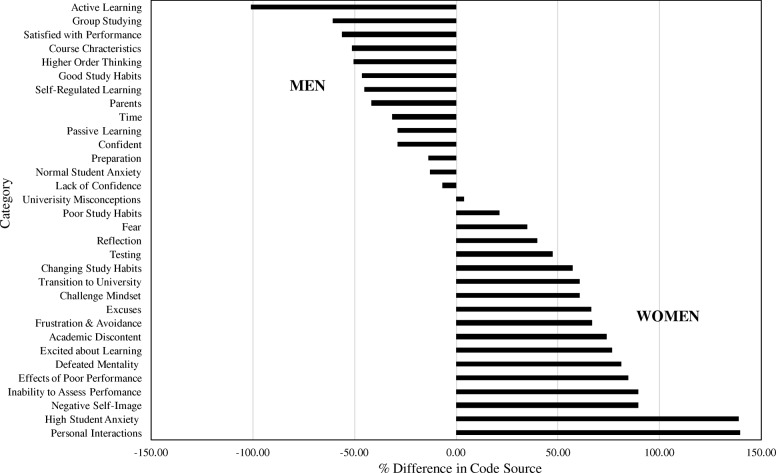
Graph showing the percent difference between the number of quotations from male and female participants for each emergent category. Percent differences were calculated relative to the number of quotations from female participants

Results of the content analysis are shown in Fig. 2. Because percent differences are reported relative to female participants, negative percentages indicate that more male students contributed statements to the focal category. A percent difference of 0 identifies categories where statements were equally likely to come from male or female participants. Positive percentages mean that more female participants contributed statements to the category in question. Male students made more statements aligned with general course characteristics or commented specifically on the course’s emphasis on active learning and group studying. Male participants also contributed more statements expressing satisfaction with their course performance. Male and female students were equally as likely to make statements aligned with categories such as University Misconceptions, Poor Study Habits, and Fear. Many of the categories dominated by statements from female students are found within the theme of Negative Academic Emotions. Female students were also more likely to make statements describing challenges coming to the university (i.e., Transition to University) and to describe the use of poor self-regulated strategies. Female students overwhelmingly made more statements representative of High Student Anxiety despite several male participants in the study reporting a high CTAS score. Statements about the importance of social and professional connections (i.e., Personal Interactions category) came almost exclusively from females.

## Discussion

The emergent model and gender analysis presented here are grounded in students’ descriptions of their university experiences. It builds on previous studies by offering a more comprehensive representation of how academic emotions could influence achievement, as well as how the gendered gap in student anxiety relates to other academic emotions. Our model confirms aspects of the relationship between emotions and performance described in (Pekrun et al. 2014, 2002) as well as the relationship between motivation and study habits provided in Wäschle et al. (2014). It also creates a broader interpretive framework by incorporating a wider range of academic emotions into the self-deprecating cycle proposed in Covington (1985). In doing so, it provides a more detailed picture of the types of emotions that can influence a student’s propensity to become trapped within a self-deprecating cycle of poor course performance and suggests how student anxiety can impact individuals outside of the testing environment. Additionally, the content analysis summarized in Fig. 2 reveals the stark emotional differences between male and female students’ descriptions of their university experiences. The model shows that many of the statements representing negative emotions are more likely to originate from female students.

### Distinctions between high and normal student anxiety

In this model, there are crucial distinctions between normal student anxiety and high student anxiety. Consistent with Pekrun et al. (2014), Chin et al. (2017), and Clark and Watson (1991), the High Student Anxiety category is centrally located among emotions, perceptions, and behaviors that hinder learning—and is much more frequently represented in the talk from female participants. This result suggests that high student anxiety is related to poor performance and academic discontent and are part of a complex network of negative emotions. For example, High Student Anxiety frequently co-occurred with categories related to how students perceived their worth or ability (Fig. 1). Examples of these type of student statements are “I’m going to fail…,” “I feel I’ve already lost the game to the other students,” and “I’d get really down and sit in my room and just blast music and be like [*sic*] you are a failure.” Highly anxious students were also more likely to bring up topics that fell under the category of Frustration and Avoidance—for example, stating that they would not attend study groups or a professor’s office hours in order to avoid negative academic emotions. These data suggest a causal link between the emotions associated with high student anxiety and detrimental behavior such as avoiding beneficial learning opportunities. Highly anxious students are also more likely to voice academic excuses to explain why they felt a certain way or why they continued to perform poorly in their classes.

It is important to reiterate that anxiety is a normal part of the human experience and that some student statements about anxiety were not always negative. Results of this study showed that normal student anxiety, in contrast, was associated with some aspects of the challenge mindset documented by Dweck and colleagues (e.g., Yeager et al. 2013), and these statements usually came from male participants regardless of their self-reported test anxiety. A challenge mindset perceives intelligence as something that can be acquired over time; conversely, a fixed mindset perceives intelligence as determined and immutable (Dweck 1986). In this study, statements in the Normal Student Anxiety category almost always focused on the actual testing event. These statements were distinguished from the High Student Anxiety Category because they were rarely associated with student talk interpreted to represent a detrimental impact on behaviors or out-of-class relationships. For example, “I’m nervous because it’s a test but in my head I’m like I’ve got this…,” “If there’s a couple questions, I feel a little nervous; but I skip it and come back…,” and “I get little bursts [during an exam] of being stressed and then I get over it.” Because previous research has shown that a challenge mindset promotes improved learning and perseverance, student talk associated with this category was inferred as part of the Positive Emotions theme. Statements that indicated a positive outlook on academics and an ability to persevere include: “I’m positive that I did well or I just get over it rather than lingering,” “I don’t want to make excuses for myself,” and “I like that these things challenge me.” Students with normal anxiety either accepted their anxious emotions as temporary or transient or framed it as a motivating influence. This interpretation is consistent with Chin et al. (2017) who reported a positive correlation between feelings of tension from test anxiety and exam performance.

Emotional resilience is often associated with high EI, and while female students are more likely to self-report higher EI than their male peers, male students in our sample were making more statements that could be interpreted as representing high EI. This was an unexpected and contradictory conclusion based on prior studies examining emotions and EI. While female students could have higher self-reported EI, many of the female students in this study described emotions, scenarios, and contexts consistent with high student anxiety and challenges in handling their emotions. Results of this study also showed that female students were quite aware of their emotions; however, due to their highly anxious thoughts and feelings—as well as their low self-efficacy and negative self-image—these students seemed to lack the emotional self-regulation typical of high emotional intelligence. These results can be interpreted to suggest that high student anxiety can impede or suppress the positive benefits of heightened EI. This is consistent with prior research showing that high anxiety can interfere with emotional regulation (i.e., Fischer et al. 2010).

In line with similar results from previous studies (Pekrun et al. 2007; Pekrun et al. 2002), positive academic emotions in our model were epitomized by student talk about being excited about learning, maintaining their confidence, and being satisfied about their performance. It is important to note that students with both high anxiety and normal anxiety discussed having similar feelings during exams. However, my results suggest that the distinction between the two categories lies in the persistence, interpretation, and self-professed consequences of the emotions.

### Emotion driving a self-deprecating cycle

This model expands on the concept of the self-deprecating cycle described by Covington (1985) and the virtuous and vicious cycles presented by Wäschle et al. (2014) by relating students’ own descriptions of poor performance and negative academic emotions to high levels of anxiety, which in turn impacts their learning strategies and reactions to poor performance. The emergent model in this study suggests that a student’s emotional response(s) to academic situations may also be a powerful factor determining study habits and self-regulation. Consequently, connections established in this model suggest that improving a student’s awareness and use of self-regulatory strategies may improve their emotional responses and study habits—providing a different approach to reducing the impact of high student anxiety.

Despite multiple experiences with poor performance, students interviewed in this study stated little desire to engage in behaviors aimed at employing self-regulated learning and improving their study habits. Several students who made statements situated in the Negative Academic Emotions theme were keenly aware of their own inadequate learning strategies but still voiced little desire to change. For example, one student stated that “exam after exam I would do the same thing,” referring to her cycle of poor studying and poor performance. Another student said “After I took the exam, I say to myself, ‘I’m going to do better for the next one. I’ve got to study really hard for the next one. And then, when the time comes, I study the same way…” and “I realized how terrible it is [referring to her study habits], but I don’t do anything to fix it.” These results are not surprising, given previous work showing that students will adopt and maintain poor learning strategies in an effort to avoid negative emotions, specifically, feelings of inadequacy or incompetence created by practicing with difficult problems instead of reviewing easier material (Lukes and McConnell 2014).

The are several parallels between the Negative Academic Emotions theme in the emergent model and aspects of the Negative Affect dimension of the TME. Both my model and the TME placed negative emotionality as a critical emotional factor and make clear distinctions between the influence of negative and positive emotions, while also considering a certain degree of overlap between them. While the types of emotions that can be considered “negative affect” are broad (Clark and Watson 1991), this study revealed through student talk that negative academic emotions centered on identity, self-efficacy, and dealing with poor performance are more associated with high student anxiety. Consistent with prior work (i.e., Chin et al. 2017), these results can be interpreted to suggest that self-deprecation driven by negative emotions related to academics is a predominant driving force behind high student anxiety.

### Self-regulated learning

Our model established through student talk that emotion plays a key role in how students employ self-regulatory strategies, in addition to how they experience and interpret anxiety. This result is consistent with prior quantitative studies showing that negative academic emotions are associated with the use of poor self-regulatory strategies (Mega et al. 2014; Pekrun et al. 2002). In addition, our data indicate that this association is reinforced by student anxiety, as the highly anxious students we interviewed evinced poor study strategies and rarely mentioned any consistent use of self-regulated learning.

Statements interpreted as aspects of students’ self-regulated learning created a central theme in the emergent model. Self-regulated learning is well-studied and describes actions and processes that are focused on the acquisition of information and skills (Zimmerman 1990). Student talk on this theme focused on self-evaluation, rehearsal, and organizational strategies. For example, students stated, “I go over the slides and talk about them, and sometimes have a bigger study group go over it,” “we get all these packets of study questions for each week, and we do those independently and then go over them together,” or “I study for bio every day. I like that. I like how I do that.” More successful and confident students would discuss strategies aligned with monitoring strategies, information seeking, and seeking social assistance. They would also make more statements about being satisfied with their academic performance and rarely used terms or described scenarios associated with high student anxiety. In contrast, most of the statements in the categories Transition to University and Inability to Assess Performance indicated poor skill-acquisition and study habits—such as continuing practices that were successful in high school—and thus negative self-regulated learning.

### Similarities and differences between male and female participants

Female students more often made statements aligned with the categories High Student Anxiety, Negative Self-Image, and Personal Interactions (Fig. 2). Male students made more statements aligned with aspects of the course design and being Satisfied with Performance. This result can be interpreted to suggest that female students are more likely to experience, or at least express, negative issues related to high student anxiety that influences their self-images and confidence. If so, the consequences towards STEM diversity could be significant. Because many of the categories in the Negative Academic Emotions theme involve poor self-perception, female students may be more likely than their male peers to become trapped in a self-deprecating cycle. Becoming trapped in a cycle of self-deprecation could reduce an individual’s confidence and motivation—which according to the model of persistence proposed by Graham et al. (2013)—can increase that individual’s likelihood of not completing a STEM degree.

The way that female and male participants viewed their academic experiences was consistent with previous studies focused on gender-based differences in test anxiety and connected them to gender-based differences in confidence and self-efficacy (e.g., Goetz et al. 2013). Data from this study indicate that the issues experienced by highly test-anxious female students often went beyond evaluative situations. Additionally, female participants also cited problems interacting with faculty and peer study groups, due to their student anxiety. Female students were also more likely to talk about avoiding situations where they might experience negative academic emotions.

Rather than talking about the emotional aspects of their undergraduate experience, male participants tended to focus on course characteristics, learning styles, and how they were accepting of their performance. At first glance, this result could suggest that male students are less “emotional” and more resilient than their female peers. However, analysis of these data in concert with my memorandum and interview reflections revealed an interesting trend in the male participants’ talk. Throughout the interviews, male students would often implicitly or explicitly avoid discussing their emotions related to academics regardless of their self-reported cognitive test anxiety and this was reflected in the content analysis of the emergent model (Fig. 2). Studies have shown that men are less likely to receive positive feedback from expressing emotions than women (McLean and Anderson 2009). This social conditioning towards avoiding conversations about their emotions could have played a part in the apparent discrepancies I noted about male participants’ willingness to discuss their emotions. Studies have also reported that an increased degree of conformity to the male gender roles in stressful events (such as high stakes testing) is also associated with higher anxiety (Eisler and Skidmore 1987). Consequently, the results of this study could be interpreted to present some level of caution as to the usefulness of self-report instruments in characterizing the academic emotions of male students and could suggest the need for gender-specific instruments to measure male and female students and test-specific anxiety more accurately.

### Implications for educators

My model reiterates the importance of teaching students to employ self-regulatory learning strategies as both a method of preparing them for academic challenges but also as a way to help promote more positive academic emotions—thus potentially providing a pathway towards better emotional resilience and regulation. Better self-regulation during studying could be important for students who struggle with high academic anxiety. It could also prevent students from becoming trapped in a self-deprecating cycle of poor performance and heightened student anxiety fueled by poor self-image and an over-reliance on ineffective study habits—or even give students a way to exit a self-deprecating cycle.

This study shows that emotion connects student anxiety and self-regulated learning. Therefore, educating students about how to better self-regulate their learning may be a way to help them *feel* better about their studying and themselves. It may also be beneficial for STEM programs that are confronting challenges with student retention and persistence to begin educating students about effective self-regulatory strategies as early in their academic careers as possible. Instruction in effective self-regulation could be an effective way to change students’ emotions towards academics and address at least some of the problems in gendered underperformance across STEM (Matz et al. 2017).

This model also suggests that some of the challenges that students face when transitioning to a university can impact their self-regulated learning and consequently their academic emotions. Fortunately, this emergent model contains malleable areas where instructors could implement interventions targeted at reducing the challenges of a student’s transition to university. This could be accomplished by addressing common misconceptions about university-level academics and to help students better understand how to gauge their ability and assess performance—as well as the benefits to acquiring such skills. Some early intervention programs have already used this approach successfully. For example, the Biology Intensive Orientation for Students (BIOS) at Louisiana State is a pre-matriculation learning community that was developed to provide students with a preview to introductory biology and incorporates instruction on learning strategies (Wheeler and Wischusen 2014; Wischusen et al. 2011). The BIOS program has enhanced students’ ability to both regulate and gauge their own learning and they have reported improvements in students’ self-efficacy (Wheeler and Wischusen 2014). Our model suggests that programs like BIOS may be a practical approach to cultivating positive academic emotions, alleviating the negative influence of high student anxiety, and preventing students from entering a self-deprecating cycle. This work and other studies suggest that female undergraduates are more likely to become “trapped” within that negative emotional cycle. Consequently, preventing female students from entering a self-deprecating cycle could be an accessible strategy for programs struggling with gender equity.

Short psychological interventions that are designed to reduce test anxiety, such as expressive writing (Ramirez and Beilock 2011) and re-appraisal practice (Jamieson et al. 2013), may also play a role in helping students escape a self-deprecating cycle. Expressive writing asks students to respond to an open prompt about their thoughts and feelings before an exam; re-appraisal practice seeks to have students reframe the physiological symptoms of anxiety as positive events. Test anxiety overlaps with other academic emotions and is co-morbid with other forms of anxiety and depression. Therefore, efforts to help students reduce their test anxiety may influence other forms of emotional distress and lead to better performance and more positive academic emotions that in turn could facilitate productive changes in study behavior.

## Study limitations

The design of this study is consistent with previous works employing emergent grounded theory analysis, and steps were taken to validate results using member checking. However, I encountered challenges getting the majority of participants to take part in the member checking. Another important limitation of this study is the inherently diffuse boundary between negative and positive academic emotions and the nature of my analysis did not always allow me to account for this ambiguity. Another hallmark of grounded theory is often collecting new data as theory emerges. Re-interviewing students was not done in this study due to funding limitations. However, codes, code groups, and categories were re-evaluated as new questions and patterns emerged from the interview data. The severity standards used in this study were not statistically validated. The three groups (low, moderate, and high) used in this study were not intended to provide any information used in the analysis. Rather, they were used to ensure that the students chosen for interviews self-reported enough issues with test anxiety to inform the development of the model. Information from the CTAS was not used beyond participant selection and did not influence the data analysis or development of the emergent model. Additionally, I typically associated poor performance with academic discontent and good performance with satisfaction, despite this association admittedly being an oversimplification for a small number of students in this study. For example, one student in my study sample was satisfied with their below-average course performance and voiced many of the characteristics that defined the positive academic emotions theme.

## Conclusions

This study produced a robust framework that connects student anxiety and other emotions with course experiences and descriptions of study habits. It also provides a model for how the gendered gap in student anxiety is expressed in a wider framework of academic emotions. It extends prior work on academic emotions and their implications on student success and re-emphasizes the importance of identifying and addressing student anxiety to encourage academic success and reduce gender gaps in STEM. Our results expand the concept of a self-deprecating cycle by suggesting that students’ emotional reactions—including student anxiety—can perpetuate negative or positive cycles, and those same emotions can influence their willingness to engage in self-regulated study strategies. Although understanding these connections has implications for both male and female students, female students may be more susceptible to becoming trapped in a self-deprecating cycle. If so, this insight could be crucial for STEM programs seeking to broaden gender diversity. Results of this study also present some data that suggests a degree of caution when interpreting survey results from male students about their academic emotions. In addition, the model proposed in this study suggests that equipping students with the tools necessary to regulate their own learning may facilitate more positive emotional reactions to academic experience thus preventing students from entering a cycle of self-deprecation.
